# The effect of grape seed protein hydrolysate on the properties of stirred yogurt and viability of *Lactobacillus casei* in it

**DOI:** 10.1002/fsn3.2188

**Published:** 2021-02-18

**Authors:** Mahnaz Samadi Varedesara, Peiman Ariaii, Javad Hesari

**Affiliations:** ^1^ Department of Food Science & Technology Ayatolla Amoli Branch Islamic Azad University Amol Iran; ^2^ Department of Food and Technology College of Agriculture University of Tabriz Tabriz Iran

**Keywords:** alcalase, antioxidant, bioactive peptides, flavourzyme, physicochemical, probiotic yogurt

## Abstract

In this study, the effect of grape seed protein hydrolysate (GPH) on the physicochemical and sensory properties of stirred yogurt was evaluated. At first, the antioxidant properties and degree of hydrolysis (DH) of GPH were determined using the microbial protease enzymes (alcalase and flavourzyme), the results showed that alcalase enzyme can produce GPH with higher DH and antioxidant properties (*p* < .05). Also, increasing the hydrolysis time had a positive effect on these properties (*p* < .05). The DH, free radical scavenging DPPH, and ferric reducing power for GPH by alcalase at 30 min was 21.51%, 88.68%, and 0.33 μmol ferrous/ g, respectively. Therefore, this treatment was used for further experiments. In the next part, the mentioned GPH was added to the stirred yogurt with three concentrations (0.5, 1.5, and 1.5%) and physicochemical properties and viability of *Lactobacillus casei* and sensory properties were measured during 15 days of storage. The results showed that the GPH treatment had higher pH, viscosity, and texture firmness and less acidity and syneresis compared with the control sample (*p* < .05). Also, in these samples, the decreasing trend of *L. casei* viability was slower than the control treatment during the storage period (*p* < .05). In most parameters, better results were observed with increasing the concentration GPH and all the treatments were acceptable in terms of sensory properties. Therefore, by producing yogurt containing GPH, a new functional food can be provided for consumers of dairy products, which in addition to the desired taste, good nutritional properties can be also achieved from its consumption.

## Graphical abstract

Probiotic products are one of the most common types of functional foods, and in recent years, there has been an increasing effort to use probiotic microorganisms in the production of various foods, and now, probiotic yogurt is the most accepted and a widely used probiotic product in the world. According to the results of this study, the industrial production of probiotic yogurt containing hydrolyzed protein as a functional food, has a favorable taste and proper nutritional properties seems to be practical and worth further study in future studies.

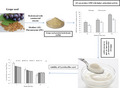

## 1 INTRODUCTION

From the distant past, yogurt has been considered because of its desirable sensory properties and significant amounts of nutrients (Ghasempour et al., 2020). Probiotic products are one of the most common types of functional foods and in recent years there has been an increasing effort to use probiotic microorganisms in the production of various foods, and now, probiotic yogurt is the most accepted and a widely used probiotic product in the world (Champagne et al., 2018; Ranjbar et al., 2020). Probiotics not only do not cause problems in the production process, but also are very effective in promoting the function of the product and improving the health of consumers (Ranjbar et al., 2020; Moghaddam et al., 2020). One of the most common microbial cultures used in the production of various probiotic products is the probiotic bacterium *Lactobacillus casei* (Asadzadeh et al., 2020). *Lactobacillus casei* is one of the most important probiotics in food products. This bacterium is gram‐positive, mesophilic, rod‐shaped, microaerophilic, catalase negative, and without spores and is attributed to the highest viability in fermented dairy products (Won et al., 2017).

Oxidation reactions in food reduce the quality of properties such as aroma, taste, color, and texture. Today, the use of bioactive peptides for food preservation, especially yogurt, is highly recommended as a substitute for synthetic antioxidants, increasing consistency, and reducing syneresis due to high water absorption (Ma et al., 2019). Bioactive peptides are protein components with potential biological activities that play an important role in promoting health (Jahanbani et al., 2016). These peptides are produced by three methods of chemical synthesis, microbial fermentation, and enzymatic hydrolysis. Among these methods, enzymatic hydrolysis is a new technique in food biotechnology, which is a controllable and gentle process and does not lead to the destruction of free amino acids. Enzymatic hydrolysis of proteins is one of the methods to improve the properties of proteins. These depend on the property of the protein and especially the type of enzyme used and the hydrolysis conditions, particularly temperature and pH (Ovissipour et al., 2013; Tkaczewska et al., 2020). One of the determining and important factors in enzymatic hydrolysis using commercial enzymes is the choice of protease enzyme. There are several types of enzymes that have been used successfully to hydrolyze food. The most important enzymes used in various studies include alcalase (which has endo‐protease activity in alkaline conditions) and flavourzyme (a mixture of endopeptidase and exopeptidase) (Nemati et al., 2012; Sinthusamran et al., 2020; Yaghoubzadeh et al., 2020). In recent years, hydrolyzed proteins with antioxidant and wholesome properties have been produced from many animal and plant sources such as milk, soybeans, wheat germ, canola, egg yolk protein, marine blood clams, oysters, and fish or shrimp waste (Chi et al., 2015). Among plant and animal sources suitable for the production of hydrolyzed protein, plant sources have received more attention due to their reasonable price and less allergenicity (Feyzi et al., 2015). Grape seed (*Vitis Vinifera*) is a by‐product of fruit juice factories. The polyphenols in grape seed include flavonoids, Gallic acid, monomeric flavan‐3 catechins, epicatechin‐3‐galite and dimeric, monomeric, and polymeric proanthocyanidins (Poiana, 2012). Currently, the use of plant protein (grape seed) has become very important due to its high nutritional value, positive effects on tissue, and reduced yogurt syneresis, as well as its positive effect on improving the growth and viability of yogurt starters, especially probiotic bacteria, during storage (Behfar et al., 2013; Sheng et al., 2016). According to the stated content, the purpose of the present study was to investigate the antioxidant properties of bioactive peptides resulting from enzymatic hydrolysis of grape seed by alcalase and flavourzyme enzymes separately on the viability of *Lactobacillus casei* and the physicochemical and sensory properties of stirred yogurt.

## 2 MATERIALS AND METHODS

### 2.1 Raw materials

Grapes used by the *Vitis Vinifera* family, Vitis genus, Euvitis subgenus, and Iranian grape species were prepared from the local market of Gilan city and approved by the Cultivation and Development Group of the Tehran Institute of Medicinal Plants. After purchasing red grapes, the seeds were removed from red grape pulp manually. After washing, the seeds were dried in a vacuum oven at 50°C for 30 min. The dried seeds were completely pulverized by shredder and packed in plastic bags of 50 g and stored at 25°C until the beginning of the experiments.

Alcalase enzyme (extracted from Bacillus licheniformis) and flavourzyme enzyme (extracted from Aspergillus oryzae) were prepared from Novozyme Company (Bagsvaerd, Denmark) and stored at 4°C until the time of being used.

Enzymatic activity was presented as Anson unit per kilogram of protein to substrate (Au/Kg protein). All chemicals used were manufactured by Merck Company (Darmstadt, Germany) and were of analytical grade.

### 2.2 Production of hydrolyzed protein

#### 2.2.1 Preparation of protein isolates from grape seeds

Grape seeds were soaked in 4% NH4OH solution (1:4 v: w) for 2 hr at 45°C in order to remove polyphenolic compounds from the seeds. This process was repeated 3 times until soaking the solution became colorless; then, the seeds were washed several times with tap water to release alkaline solutions; then, the seeds were dried in the sun. Grape seeds were pulverized using a home mill to extract protein. Lean grape seed powder was stirred in 1 Nacl mol / L solution (w: v 1:8) at ambient temperature for 30 min, and then, its pH was adjusted to 5.9 using 1NolOH mol/L. After stirring for 30 min, the suspension was centrifuged at 800rpm for 20 min at 4°C (Model Z36HK, Hamburg, Germany). The supernatant was adjusted to pH 4 using 1 Hcl mol/L to precipitate proteins and centrifuged again at 800 rpm for 4 min at 4°C. The precipitates were washed several times with distilled water and adjusted to pH 7 in distilled water containing 0.1 mol/L mol/L up. Scattered particles were dried using a freeze dryer (Operon FDB‐550, South Korea) (Karami et al., 2019).

#### 2.2.2 Hydrolysis of grape seed protein isolate

50 g of the sample was poured into a 250 ml Erlenmeyer flask, and then, 100 ml of distilled water in the ratio (2:1) was added to the Erlenmeyer flask and homogenized with a digital stirrer for 2 min. Then, the optimal pH of enzyme activity was reached (alcalase 8.5, flavourzyme 7) by adding 0.2 normal sodium hydroxide. Samples were placed in a mobile water bath at 57°C for the alcalase enzyme and 55°C for the flavourzyme enzyme to produce a hydrolyzed protein at a constant speed of 200 rpm, then the enzyme (1% of the protein content of the grape seed protein isolate) was added to it and at the end of the experiment (time 30 min), they were placed in a water bath for 15 min at 95°C to stop the enzymatic reaction. After cooling, the hydrolyzed proteins were centrifuged at a constant speed of 6,700 rpm for 20 min, the floating liquid was collected and the hydrolyzed protein was stored in the freezer, and then, it was pulverized using a freeze dryer (Ding et al., 2018).

#### 2.2.3 Degree of hydrolysis

The degree of hydrolysis was calculated based on the amount of α amino acid in the amount of sample protein (Sinthusamran et al., 2020).

### 2.3 Determination of antioxidant activity

#### 2.3.1 Measurement of antioxidant activity by DPPH method

1.5 ml of the solution sample was combined with 1.5 ml of DPPH (0.15 mM in 95% ethanol). It was read at 517 nm after 30 min of incubation in the dark and at the temperature of the adsorption medium and the control is prepared in a similar way. Distilled water was used instead of the solution sample (Yaghoubzadeh et al., 2020).

Determination of DPPH free radical scavenging activity was calculated using the following formula:

Percentage of free radical scavenging DPPH= (blank adsorption / blank adsorption–sample adsorption) ×100%

#### 2.3.2 Ferric Reducing Antioxidant Power assay measurement (FRAP)

0.5 ml of the solution was combined with 2.5 ml of 0.2 M phosphate buffer (pH 6.6) and 2.5 ml of 1% potassium ferric cyanide, and the mixture was incubated at 50°C for 20 min. And then mixed with 2.5 ml of 10% trichloroacetic acid (TCA) and centrifuged at a constant speed of 3,000 rpm for 10 min and 2.5 ml of the top layer of this solution with 2.5 ml. liters of distilled water, and 2.5 ml of 0.1% ferric chloride (Fecl3) were combined and placed at ambient temperature for 10 min, and then the absorbance was read at 700 nm (Ketnawa & Liceaga, 2017).

Based on the results of the antioxidant test, a better treatment was used for the next tests.

#### 2.3.3 Amino acid composition

The hydrolyzed protein powder was completely hydrolyzed for 24 hr at 110°C using 6 N hydrochloric acid. Then, derivatization of amino acids was performed using phenyl isothiocyanate (PITC). The amount of total amino acids was determined using HPLC model Smart line (Berlin, Germany) using column C18 with fluorescence detector (RF‐530) (Hamzeh et al., 2018).

#### 2.3.4 Preparation of functional probiotic yogurt

First, standardized milk (containing 1.5% fat and 10.5% non‐fat dry matter) was pasteurized at 90°C for 5 min. After homogenization, it was cooled to 43°C. Yogurt starter (*lactobacillus delbrueckii*, the subspecies of bulgaricus and *Streptococcus thermophilus*) was added to milk along with probiotic bacteria (*Lactobacillus casei*). At the same stage, hydrolyzed protein of grape seed was added at different levels (0.5, 1.5, and 1.5%) (Control sample was considered without adding hydrolyzed protein). Then, incubation was performed at 43°C until pH = 4.7. Then, stir for 3–5 min to get a completely uniform appearance. In the next step, the obtained product was packed in 100 g containers and then the samples were stored in the refrigerator at 4°C, and finally the tests were performed at intervals of 1, 8, and 15 days with three replications (Ghasempour et al., 2020).

#### 2.3.5 PH determination

To measure pH, a pH meter was used based on the amount of free H + ions in the sample. To measure the pH of the samples, before each operation, the pH meter was first calibrated by 2 buffers 4 and 7. PH changes were observed during incubation and the end of incubation and during the storage period in the refrigerator in three replications (Ghasempour et al., 2020).

#### 2.3.6 Measurement of acidity

Acidity was measured by titration method using burette and 0.1 N sodium hydroxide and phenolphthalein reagent. First, the yogurt sample was completely uniform; 10 grams of yogurt were removed from the container and poured into a 100 ml glass beaker. 10 ml of distilled water was added to dilute it. Then, 3–4 drops of phenolphthalein solution were added to it and titrated with 0.1 N sodium hydroxide. After the appearance of pale pink color, the volume of Sodium hydroxide consumed was read from the burette and entered in the following formula (AOAC, 2005).

TA or Acidity = $\frac{V\times 0/9}{M}$

V = volume of sodium hydroxide solution consumed in milliliters

*M* = sample weight

#### 2.3.7 Syneresis

The specified weight of the yogurt cup was kept at a 45° angle in the refrigerator for 2 hr at 5°C to separate the yogurt water. The separated water from the yogurt surface was sucked using a syringe and weighed. Syneresis is calculated as a percentage by weight of the separated yogurt water to the initial weight of yogurt (Zainoldin & Baba, 2009).

$$y=\frac{a}{b}\times 100$$

y = percentage of syneresis

a = separated yogurt water

b = yogurt initial weight

#### 2.3.8 Viscosity measurement

The apparent viscosity of the samples, based on the method of Ranadheera et al. (2012), was determined by a Brookfield viscometer. The sample temperature at the time of viscosity reading was 15°C and its volume was 100 ml. Spindle No. 2 and 0.5 rpm were used for 1 min. This test was performed for each sample in three replications. And viscosity was reported in centipoise (CP).

#### 2.3.9 Evaluation of yogurt texture

Texture hardness of yogurt samples was measured using a texture analyzer (Brockfill Company with a loadcell of 4,500 g, model LFRA‐4500). The probe used was a cylindrical type with a diameter of 38 mm. The penetration rate of the probe into the sample was 1 mm/s, and its penetration depth was selected to be 30 mm. It should be noted that the manufacturer's instructions were used to select the type of probe and other parameters used (Domagała, 2009).

#### 2.3.10 Color analysis

The samples were selected to evaluate the color parameters (L, a* and b*). Visible color parameters were measured by a Colorflex Hunter Lab colorimeter (Hunter Lab Inc., Reston, USA) (Ghasempour et al., 2020). The color parameters were L* (luminosity) for lightness, ranging from 0 for black to 100 for white, a* (redness) for red/green, and b* (yellowness) values for yellow/blue.

### 2.4 *Lactobacillus casei* counting

1 ml of the homogenized sample was mixed with 9 ml of physiological saline and made uniform and diluted to 10^9^ and 10^10^ and then 1 ml of each dilution in 3 replicates was transferred to a plate containing culture medium‐acidic MRS‐bile agar. After complete mixing, it was placed in an anaerobic jar under incubation conditions for 72 hr at 37°C. After this time, the number of *Lactobacillus casei* bacteria in the sample was determined (ISIRI, 1992).

#### 2.4.1 Yogurt sensory evaluation

Yogurt Sensory evaluation was performed using a 7‐point hedonic scale (1‐very bad and 7‐excellent). Panelists were selected from among 10 graduate students in the food industry. Features evaluated include color, taste, texture, and general acceptance. Yogurt samples were coded with numbers and randomly tested for sensory tests. All samples were sensory analyzed by panelists at 7°C (after being removed from the refrigerator for 10 min), and mineral water was used to evaluate the taste of each yogurt sample (Won et al., 2017). Test was performed in 3 replications and in all 3 replications on the first and 15th day of yogurt refrigeration time. After providing an explanation to the panelists, evaluation forms were provided to them and the panel test was done.

#### 2.4.2 Statistical analysis

All experiments were performed in a completely random experimental design with three replications, and the results were reported as mean (average) with standard deviation. Statistical analysis of treatments was performed by variance analysis table (ANOVA) using IBM SPSS Statistics 22.0 (IBM SPSS, Inc., Chicago, IL, USA). Duncan test at the level of 0.05 was used to express the significant difference between the means (averages), and the graphs were drawn with Microsoft Excel software.

## 3 RESULTS AND DISCUSSION

### 3.1 Investigation of hydrolysis degree

The degree of hydrolysis is used as a parameter to monitor the amount of protein hydrolysis; this factor is mostly used as an indicator to compare between different hydrolyzed proteins. On the other hand, the degree of hydrolysis is one of the most important factors in the study of the properties of hydrolyzed proteins, which indicates the degree of breaking of peptide bonds and should be controlled. With increasing hydrolysis time (Table 1), the degree of hydrolysis increased. This may be due to the fact that more enzymatic activity takes place over a longer period of time and the peptide bonds become available for a longer period of time and this consequently results in a higher degree of hydrolysis due to more breakings of peptide bonds. Also, the degree of hydrolysis by alcalase was higher than flavourzyme. The choice of enzyme plays a very important role in the production of hydrolyzed protein, because each enzyme has a different pattern in breaking of peptide bonds that will affect the amino acid composition, molecular weight, and bioactivity of the produced peptides (Ko et al., 2013). Alcalase is frequently used by various researchers due to the production of hydrolyzed proteins with high degree of hydrolysis in a short time (Hamzeh et al., 2018; Nemati et al., 2012; Sinthusamran et al., 2020). Similar results were reported by Sbroggio et al. (2016). They investigated the effect of hydrolysis time and type of enzyme including alcalase and flavourzyme on the degree of hydrolysis of Okara hydrolyzed protein (a low‐value by‐product of soybeans). They also stated that with increasing hydrolysis time, the degree of hydrolysis increased and the highest values of degree of hydrolysis were observed by the enzyme alcalase.

**TABLE 1:** Degree of hydrolysis of grape seed protein hydrolysate at different reaction time using different enzymes ^a^, ^b^

Hydrolysis time(min)	Enzymes	
	Alcalase	Flavourzyme
10	11.09 ± 0.14^Ac^	8.38 ± 0.38^Bc^
20	17.54 ± 0.51^b^	12.65 ± 0.58^b^
30	21.51 ± 0.55^b^	15.80 ± 1.04^b^

^a^ Values represent means ± SE (*n* = 3).

^b^ Values in same columns with different superscripts are significantly different at *p* <.05.

### 3.2 Antioxidant properties

#### 3.2.1 DPPH free radical activity

**FIGURE 1:**
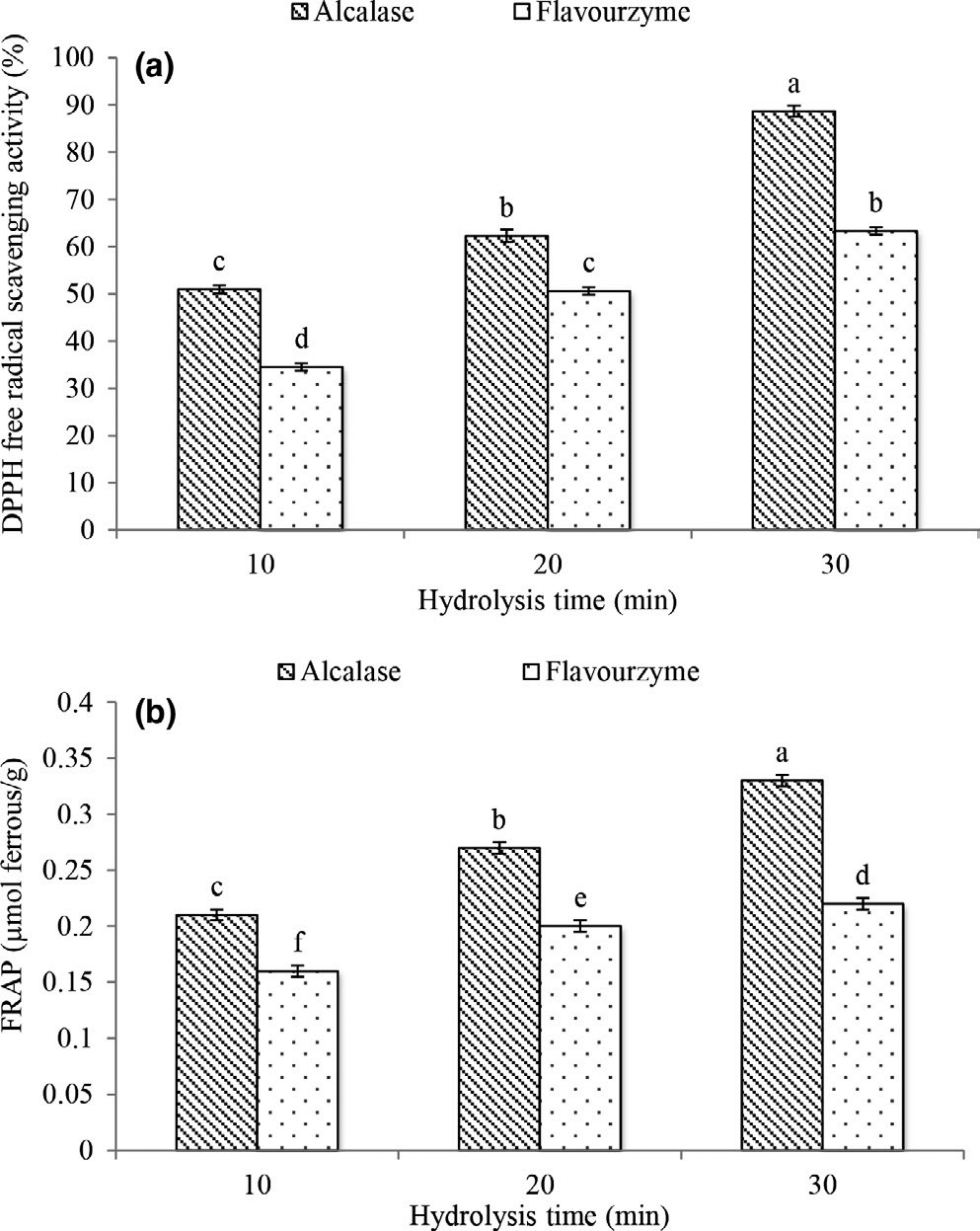
The antioxidant activity [DPPH (a) and FRAP (b)] of grape seed protein hydrolysate (GSH)

The DPPH free radical activity test is commonly used to measure the free radical scavenging ability of a sample. DPPH is a lipophilic radical with a maximum absorption at 517 nm that tends to accept protons, thus When a free proton compound is present, such as an antioxidant, it reduces the absorption measurement by DPPH free radical scavenging. Therefore, the antioxidant property of such a compound is expressed as its ability to DPPH free radical scavenging (Bagheri et al., 2016; Javadian et al., 2017). The results of the present study showed (Figure 1a) that all hydrolyzed proteins had a high potential for DPPH free radical scavenging. The bioactive peptides in hydrolyzed proteins exert their antioxidant activity through various mechanisms such as inhibition of lipid peroxidation, free radicals scavenging, and chelation of metal ions (Correa et al., 2011). DPPH free radical scavenging in hydrolyzed protein of other plant proteins such as carrot seed (Ye et al., 2018), wheat germ (Karami et al., 2019), oil turmeric (*Moringa oleifera*) (Aderinola et al., 2018) Quinoa seed (Mahdavi‐Yekta et al., 2019), and Alternanthera (*Amaranthus cruentus*) (Ramkisson et al., 2020) were also announced.

It is believed that the type of raw material, enzyme specificity, hydrolysis conditions and size, amount and structure of amino acids, and peptides produced are considered as effective factors on biological activities (Kamau & Lu, 2011). The results of this study showed that hydrolysis conditions affect the antioxidant activity of grape seed and the antioxidant activity of protein hydrolyzed by alcalase enzyme was higher. In order to produce antioxidant peptides during protein hydrolysis, the enzyme must be able to hydrolyze specific peptide bonds in the protein chain, which in this study, alcalase was able to create better peptide bonds (De Castro & Sato, 2015). These results are consistent with the results of Mahdavi‐Yekta et al., (2019). They also reported that with increasing hydrolysis time (up to 150 min), the amount of antioxidant activity of quinoa seed hydrolyzed protein increased.

Hydrolysis Independent variables have a direct effect on enzyme activity and consequently on the antioxidant properties of the final peptides. Therefore, evaluating the hydrolyzed antioxidant potential by performing more than one type of method provides a better understanding of their activity.

#### 3.2.2 Ferric reducing antioxidant power

Measuring method of ferric reducing antioxidant power evaluates the electron donation potential of an antioxidant compound such as peptides, which reduces Fe^3 +^ to Fe^2 +^ ions. The antioxidant potential of a bioactive compound is directly related to its reducing power, which has been confirmed in previous studies (Aderinola et al., 2018).

The results of the present study showed (Figure 1b) that all hydrolyzed proteins had a high ability to reduce iron. They had the ability to reduce iron in the hydrolyzed protein of other plant proteins, and it was also announced in plants such as carrot seeds (Ye et al., 2018), oil turmeric (*Moringa oleifera*) (Aderinola et al., 2018), and Alternanthera (*Amaranthus cruentus*) (Ramkisson et al., 2020).

Reducing antioxidant power of ferric ion of protein hydrolyzed by alcalase enzyme was higher. Also, as the hydrolysis time increased, the amounts of Ferric ion reducing power increased. Antioxidant activity of protein hydrolyzed by alcalase enzyme with 30 min had the highest antioxidant activity (0.33 μmol ferrous/g). The difference in ferric reducing power between proteins hydrolyzed by different enzymes can be attributed to the presence of specific peptides with specific amino acid sequences and specific molecular weights. For example, it has been reported that the antioxidant activity of peptides with a molecular weight of 500 to 1,500 Daltons is stronger than peptides with a molecular weight higher than the molecular weight of 1,500 and peptides below 500 Daltons (Sheng et al., 2016). By increasing the hydrolysis time and decreasing the size of the produced peptides, the ability of peptides to remove free radicals was increased.

#### 3.2.3 Amino acid composition

Results related to amino acid levels of protein hydrolyzed by alcalase after 30 min of hydrolysis showed (best treatment in previous tests) (Table 2). The highest amino acid levels in the present study were the non‐essential valine amino acid 7.98% and then the leucine amino acid 7.11%, and the glycine amino acid 12.55%. Ding et al. (2018) stated threonine, valine, and leucine as the highest levels of essential amino acids in hydrolyzed grape seed protein, and glutamic acid, proline, and glycine as the non‐essential amino acids which were consistent with most of the amino acids in the present study. Amino acids neutralize free radicals by proton donation. Fat‐soluble free radicals (peroxyl radicals) produced throughout the oxidation of unsaturated fatty acids are neutralized by hydrophobic amino acids such as leucine, valine, alanine, and proline (Rabiei et al., 2019). Therefore, it can be argued that hydrolyzed proteins may have inhibitory effects on several types of free radicals due to the presence of different amino acids.

**TABLE 2:** The amino acid composition of grape seed protein hydrolysate (g 100 g^‐1^) (30 min)

Amino acid(g 100 g^−1^)	Alcalase
Histidine ^a^	4.25
Isoleucine ^a^	2.98
Leucine^a^	7.11
Lysine^a^	2.16
Methionine^a^	1.05
Phenyl alanine^a^	2.45
Tyrosine	4.59
Threonine^a^	6.98
Arginine^a^	5.98
Valine^a^	7.98
Aspartic acid	10.25
Glycine	12.55
Proline	2.98
Serine	3.94
Alanine	1.95
Cystein	1.09
Glutamic acid	19.55
Total amino acid	98.85
Essential amino acid/non‐essential amino acid	1.74
Essential amino acid/total amino acid	63.62

^a^ Essential amino acids.

According to FAO/WHO (1990), the ratio of essential amino acid to total amino acids should not be less than 40% and also the amount of essential to non‐essential amino acids should not be less than 0.6. According to the results, the hydrolyzed protein has a suitable amino acid composition. The ratio of essential to non‐essential amino acids is 1.74, and the amount of essential amino acids to the total available amino acids is 63.62.

#### 3.2.4 PH and acidity values of yogurt

PH is a logarithmic quantity that expresses the ionic strength of hydrogen in aqueous solutions and is actually a measure of the degree of acidity or alkalinity of aqueous solutions.

In terms of dairy and yogurt production standards, measuring acidity is one of the most important tests and will increase its shelf life and better acceptance by the consumer. Acidity is the main characteristic of lactic acid, which is produced by the fermentation of lactose and is considered as one of the important tests and also one of the acceptable indicators for yogurt storage (Yilmaz & Kurdal, 2014).

**FIGURE 2:**
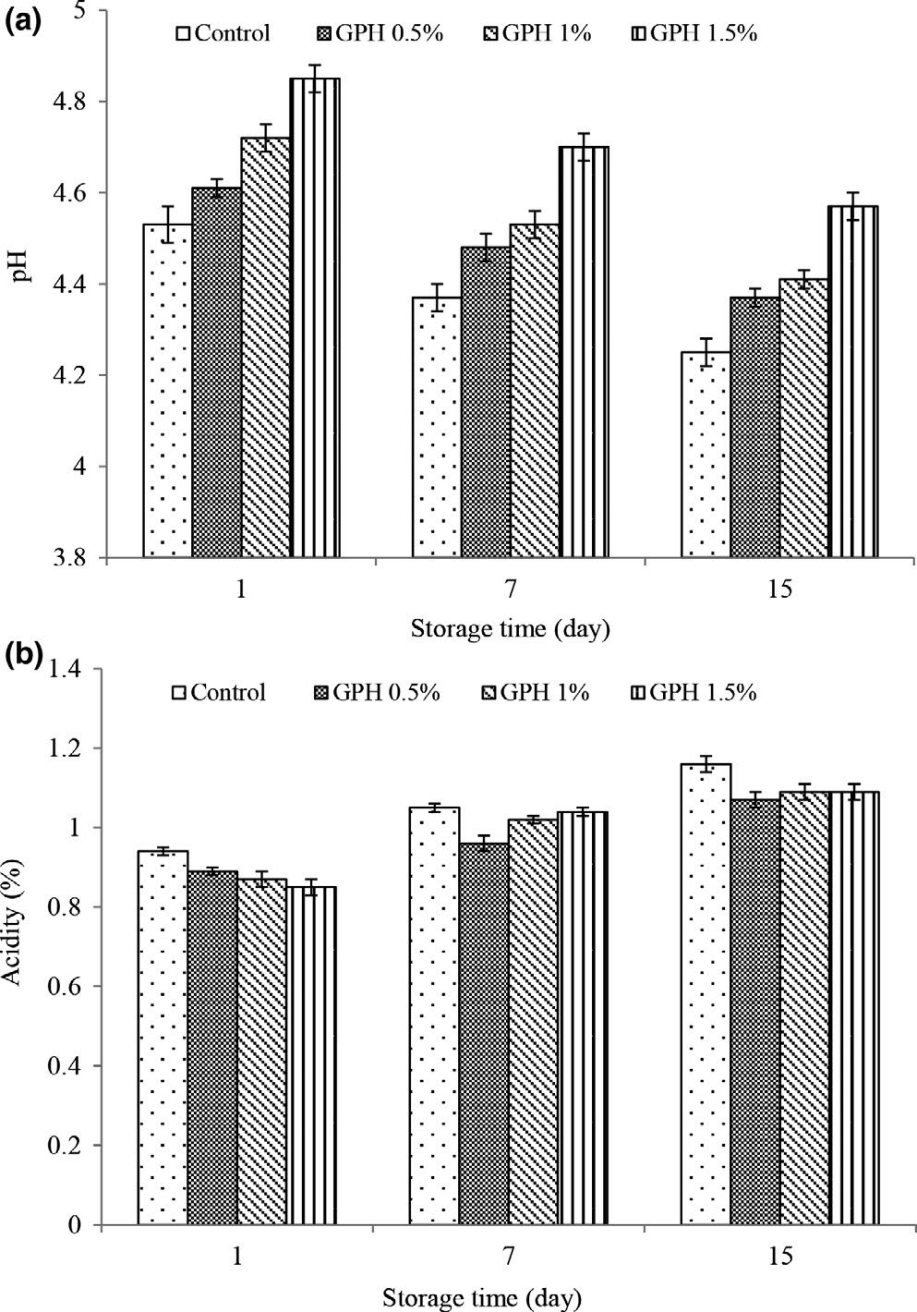
Changes in pH (a) and acidity (b) of different treatment during storage

The results of changes in pH and acidity (Figure 2a,b respectively) during the maintenance process showed that pH values decreased over time in all treatments and acidity increased. During the production and storage of yogurt, the catabolism of lactose by the starter bacteria causes the production of lactic acid and increases the acidity and decreases the pH. The trend of decreasing pH and increasing acidity during storage is to be expected, which has been reported in most related studies (Ma et al., 2019; Chi et al, 2015).

The results related to the pH and acidity of yogurt values showed that the addition of hydrolyzed grape seed protein increased the pH and decreased the acidity, and in all storage times, the highest pH and lowest acidity values were observed in the treatment containing 1.5% hydrolyzed protein. This is due to the buffering properties of hydrolyzed proteins and minimizing yogurt acidity during storage (Marta Henriquesa et al., 2011).

#### 3.2.5 Yogurt syneresis values

**FIGURE 3:**
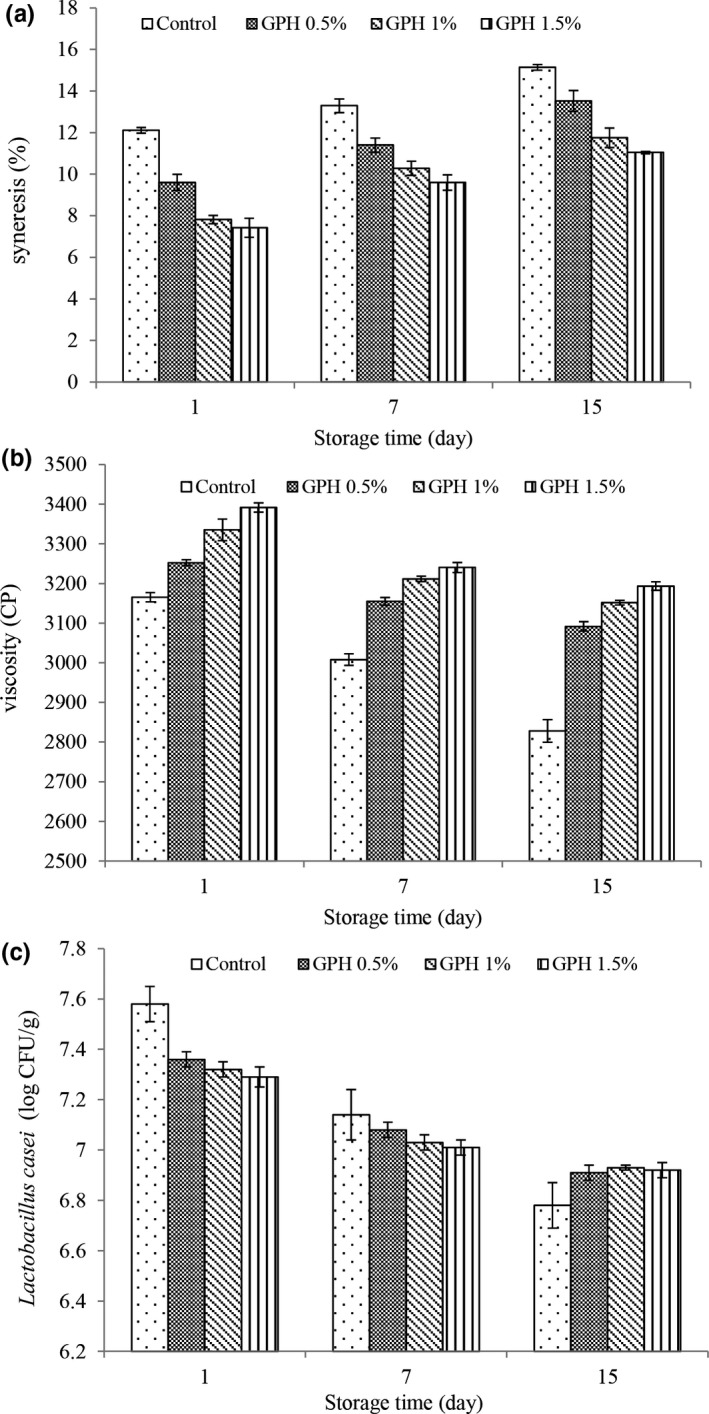
Changes in syneresis (a), viscosity (b), and *Lactobacillus casei* (c) of different treatment during storage

Measuring the value of synergy is one of the most important physical tests to measure the quality of yogurt. Gel synersis is the separation of the aqueous phase from the continuous phase, that is, the gel network. This phenomenon is favorable in cheese making and unfavorable in yogurt production. Synersis values (Figure 3a) increased over time in all treatments. The phenomenon that occurred during synersis is not fully understood, but it is agreed that increased synersis with maintenance time is associated with severe rearrangements of the casein network that increase serum outflow, rearrangements lead to an increase in particle binding, so the network tends to compress and shrink, resulting in the separation of serum (Ramirz‐Santiago et al., 2010). Synersis seems to be largely related to the amount of casein in milk or the addition of stabilizers. With the addition of hydrolyzed protein, the synersis values of yogurt were significantly reduced, which is due to the increase in dry matter and also the water absorption capacity of hydrolyzed protein. Dabija et al. (2018) also reported that adding natural protein of hemp to yogurt reduces synersis and increases the storage capacity of low‐fat yogurt.

#### 3.2.6 Yogurt viscosity values

Yogurt viscosity is an important property that affects its quality. Viscosity is affected by various factors such as incubation temperature, dry matter content, heat treatment of milk, milk acidity, and type of starter culture. According to the results, viscosity values (Figure 3b) decreased over time in all treatments (*p* <.05). The decrease in viscosity during the storage period can be due to changes in the protein–protein binding in the three‐dimensional protein network of yogurt samples (Boeneke & Aryana, 2008). In the present study, by adding hydrolyzed protein, the viscosity increased compared with the control treatment. This may be due to the increase in dry matter and the binding of the added protein to the free water and the decrease in flowability, the involvement of the casein network and the increase in the sample resistance to leakage (Sakandar et al., 2014). Dabija et al. (2018) also stated that adding natural protein of hemp to yogurt increases the viscosity of yogurt.

#### 3.2.7 Yogurt firmness values

Firmness index indicates the maximum force in the compression state, which is one of the important properties of yogurt texture. Yogurt texture firmness (Figure 3c) decreased significantly with increasing time in all treatments and the firmness values in the treatments containing hydrolyzed protein of grape seed at different times were significantly higher than control treatment and in all storage times (*p* <.05), and the highest firmness values were observed in the treatment containing hydrolyzed protein 1.5% (*p* <.05). The firmness of yogurt texture depends entirely on the dry matter of the product, the amount of fat, and especially its protein. Increasing the amount of dry matter and protein, by increasing the amount of cross‐linking in yogurt gel network, leads to the formation of a three‐dimensional network and stronger gel structure. High protein content in the samples causes cross‐linking, followed by the formation of a three‐dimensional protein network and stronger gel structure in the produced samples; therefore, the lower firmness of yogurt texture in the control sample may be due to the lower protein content compared with other samples. On the other hand, excessive increase in texture firmness affects the sensory properties of yogurt samples and causes the aromatic substances in yogurt cannot be released in the mouth (Akin & Ozcan, 2017).

#### 3.2.8 Viability of the bacterium *Lactobacillus casei*

According to the recommendation of the International Dairy Federation, the amount of live and active probiotic bacteria by the end of the product storage period should be at least 10^7^ cfu / ml. However, this number is mentioned in the Iranian International Standard 10^6^ cfu / ml and in some other sources 10^8^–10^9^ cfu / ml (Sahadeva et al., 2011). Viability rates of *Lactobacillus casei* (Figure 3c) decreased in all treatments with increasing storage time. The highest viability rates of *Lactobacillus casei* were observed at the beginning of the storage period in the control treatment and at the end of the storage period were observed in treatments containing hydrolyzed protein. In these treatments, the levels of *Lactobacillus casei* were about 10^7^ cfu / ml. Moreover, at the end of the storage period, the lowest values were observed in the control treatment. Most studies indicate low viability of probiotic in yogurt. These researchers stated that high acidity and relatively low pH of yogurt are the main reasons for the decrease in the number of probiotic bacteria during storage (Sahadeva et al., 2011). According to the argument made by these researchers, the higher viability of *Lactobacillus casei* in treatments containing hydrolyzed protein may be due to higher pH and lower acidity in these treatments.

#### 3.2.9 Yogurt color index values

**FIGURE 4:**
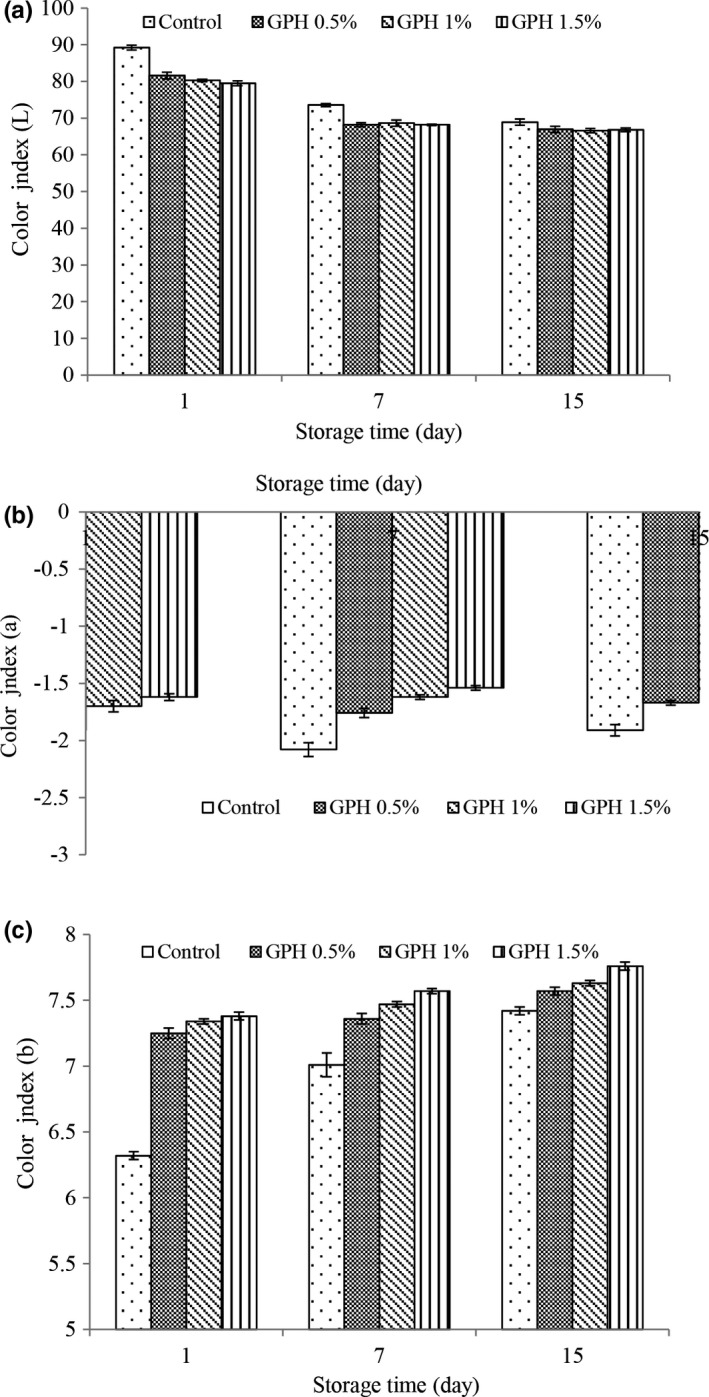
Changes in color index L (a), color index a (b), and color index b (c) of different treatment during storage

Color is one of the most important visual properties in food that changes in physical, chemical and microbial properties of yogurt affect its shelf life and may cause the destruction of this property. The results of color index (Figure 4) of the present study showed that by adding hydrolyzed protein to yogurt at the beginning of the storage period, the values of color index L decreased and the values of color index a and b increased, which is due to the yellowish color of hydrolyzed protein that reduces the brightness and increases the yellowness and redness of yogurt. With increasing storage time, the values of color index L in all treatments decreased and the values of color index a and b increased. Garcia et al. (2005) stated that color indices correlate with pH, so that lowering the pH during storage can reduce brightness and increase redness and yellowness. Proteolysis of yogurt fat during storage by microbial protease enzymes can also be a factor in reducing the brightness of yogurt during storage. These factors may be the reason for the decrease in the brightness of the yogurt and the increase in redness and yellowness observed in the examined yogurt during storage. Changes in color indices during storage in treatments containing hydrolyzed protein were less than the control treatment. As mentioned above, one of the reasons for this is the higher pH values in treatments containing hydrolyzed protein, as well as the antimicrobial and antioxidant properties of hydrolyzed protein have been reported in the studies of many researchers (Correa et al., 2011; Yaghoubzadeh et al., 2020). It seems that hydrolyzed protein can keep yogurt proteins out of the reach of lactic acid bacteria and reduce proteolysis by increasing the pH values and decreasing the acidity of yogurt.

#### 3.2.10 Evaluation of yogurt sensory properties

**FIGURE 5:**
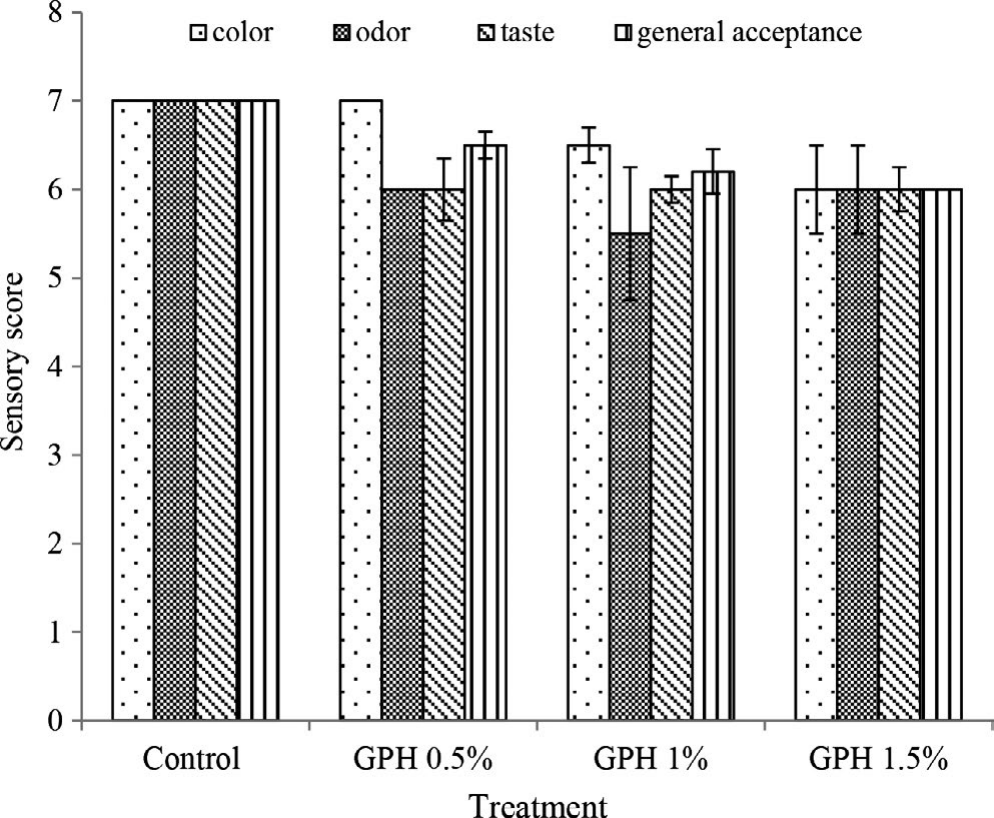
Sensory evaluation in different treatments at the beginning of storage

Sensory properties are the main factors in accepting or rejecting many products and taking satisfaction from their consumption. In the present study, sensory analysis was determined based on color, odor, taste, and general acceptance. According to the results of statistical analysis (Figure 5), the sensory score decreased by adding hydrolyzed protein and with increasing protein concentration a lower sensory score was observed, but all treatments were approved by the evaluators. The color change in yogurt containing different percentages of protein in the present study seems to be related to the color of the hydrolyzed protein powder.

## 4 CONCLUSION

According to the results of the present study, the addition of hydrolyzed protein to yogurt affects its physicochemical properties. Samples containing hydrolyzed protein had higher pH, viscosity and firmness of texture, and lower acidity and synergy compared with the control sample; in addition, it increased the viability of *Lactobacillus casei* during the maintenance period compared with the control treatment. Despite the positive effect of hydrolyzed protein on yogurt physicochemical properties, these treatments were acceptable in terms of sensory evaluations. Therefore, the industrial production of yogurt containing hydrolyzed protein as a functional food, which has a favorable taste and proper nutritional properties seems to be practical and worth further study in future studies.
